# Imaging Advances in Colorectal Cancer

**DOI:** 10.1007/s11888-016-0321-x

**Published:** 2016-04-27

**Authors:** Svetlana Balyasnikova, Gina Brown

**Affiliations:** Colorectal Imaging Group, The Royal Marsden Hospital, NHS Foundation Trust, Downs Road, Sutton, Surrey, SM2 5PT UK; Imperial College London, London, SW7 2AZ UK; The N. N. Blokhin Russian Cancer Research Center, Kashirskoye Shosse 24, Moscow, 15478 Russia; The State Scientific Center of Coloproctology, ul. Saliama Adilia 2, Moscow, 123423 Russia

**Keywords:** Rectal cancer, Staging, Imaging biomarkers, Extramural spread, EMVI, CRM, TRG, MR-defined surgical planes, Mucinous tumours, Early rectal cancer, Beyond TME, Low rectal cancer

## Abstract

The optimal management of rectal cancer is achieved through a shared multidisciplinary decision making process with accurate staging by imaging being critical for treatment planning. Good quality, high-resolution MRI has become the imaging gold standard as it allows consistent staging and stratification of patients into distinct prognostic groups according to MR-findings. Imaging features other than T and N have been proven to influence patient outcomes, and increasingly these features are taken into consideration when determining treatment options: distance of tumour to the potential circumferential margin (CRM), presence of tumour within the extramural rectal vessels (EMVI), discontinuous tumour deposits (N1c), relationship to the intersphincteric plane in low rectal tumours and to pelvic compartments in advanced disease. The presence or absence of proven adverse MR features should be included in the MRI report and shared with the patient when treatment choices are offered. MRI enables the identification of high risk tumours where the use of neoadjuvant therapy is justified and is a robust method of identifying patients with a strong likelihood of complete response after preoperative treatment.

## Introduction

Imaging plays a major role in treatment decision making of rectal cancer patients. The concept of tumour-spread assessment has markedly changed during the last decade. Rather than just T and N stage, other imaging prognostic factors and imaging-based assessment of the surgical planes can help the colorectal teams make decisions to improve outcomes (key MR imaging features are listed in the Table 1).

**Table 1:** A list of important MR imaging features

Imaging criterion	Key points
Extramural tumour spread (Fig. 1a)	For both colon and rectal cancer, extend of tumour spread beyond the muscularis propria should be measured (in mm) at the level of advanced invasion border and staged as <1 mm (T3a), 1–5 mm (T3b), 5–15 mm (T3c) and >15 mm (T3d).
mrCRM (Fig. 1b)	Minimal tumour distance to the TME plane (mrCRM) should be measured; if clearance is less than 1 mm then the potential TME plane CRM is considered involved.
Lymph nodes/vascular deposits (Fig. 1c)	Seems to be of no prognostic importance for local recurrence; N1c (tumour/vascular deposits) is of more concern and linked with extramural vascular invasion.
mrEMVI (Fig. 1c)	Large vein extramural vascular invasion should be reported on both pre- and post-CRT scans and feedback to pathologists to aid their assessment of the specimen.
Mucinous tumours (Fig. 1d)	Mucin component is readily identified on high-resolution MRIs. MR evidence of mucin within the tumour should be reported.
Tumour response assessment (Fig. 1e, f)	No uniform threshold for MR RECIST and volumetric analysis. mrTRG is proven to be an independent prognostic factor. It is reproducible and enables to identify complete responders. No validated data concerning the added value of DWI or PET/CT.
Early rectal cancer (Fig. 1g)	High-resolution MRI is accurate in staging early rectal cancer and allows identifying patients eligible for local excision.
Low rectal cancers (Fig. 1h)	Tumour distance from the anal verge and intersphincteric plane status should always be reported.
Beyond TME (Fig. 1i)	High-resolution MRI defines the safe surgical planes. Every pelvic compartment should be assessed for tumour spread.

Type of surgery to be performed mainly depends on an accurate assessment of the local extent of tumour. Screening programmes are currently identifying more early rectal tumours and potentially benign polyps. However, in up to 30 %, these appear to be more aggressive on final histology after local excision [1]. When selecting patients for local excision surgery the purpose of staging is not just to exclude the presence of malignant lymph nodes but also to assess the depth of invasion and the degree of preserved submucosa and muscularis propria to enable a definitive excision procedure to be undertaken without the risk of an involved resection margin.

For locally advanced rectal cancers, preoperative chemoradiotherapy (CRT) is a standard of treatment. The term ‘locally advanced’ and its definition varies between centres and countries. The avoidance of preoperative therapy in patients with a low risk of local recurrence or distant failure is gaining widespread acceptance in Europe but is based on outcome data that relates to audited surgical total mesorectal excision (TME) with removal of tumour and draining nodes in a single ‘package’. There is also widespread agreement that regardless of T and N stage, tumour extending to within 1 mm of the surgical TME plane (mesorectal fascia and intersphincteric plane) is associated with a high risk of local recurrence. Such patients are accurately identified by MRI and offered preoperative chemoradiotherapy [2–4]. MRI findings of positive extramural venous invasion (EMVI) status, presence of mucin component within the tumour and invasion of the intersphincteric space in low rectal tumour are also proven risk factors for poor outcome [5••, 6]. In primary rectal cancer patients with TME planes involved, long-term results are better if radical exenteration surgery is applied in the first instance [7, 8] compared to those receiving exenteration for recurrent disease [9, 10]. Therefore, imaging is needed for defining surgical planes and identifying necessity of preoperative treatment rather than just assessing tumour spread by TNM classification.

Stage-directed treatment plan based on pretreatment staging of rectal cancer is recommended by the National Comprehensive Cancer Network (NCCN) guidelines [4]. Clinical trials are underway to determine whether reassessment of the tumour after preoperative CRT by high resolution MRI enables a change of surgical planes in rectal cancer patients. Formal assessment of shrinkage and downstage of the primary tumour is needed to determine if an altered strategy following treatment is safe and improves sphincter preservation rates (TRIGGER: EudraCT Number 2015-003009-40). Approximately 30 % of patients show complete response on final pathology and theoretically could have had their surgery omitted—thus the role of imaging in identifying such patients following completion of chemoradiotherapy is also being evaluated in a randomised clinical trial (EudraCT Number 2015-003009-40) [11]. Currently centres use assessment by sigmoidoscopy/endoscopy but lack sensitivity in finding patients with complete response; therefore, new ways of differentiating changes within a treated tumour, through MRI would enable the possibility of organ preservation in many more patients than currently achieved [12].

## Prognostically Important Imaging Biomarkers

### Extent of Rectal Tumour Within the Mesorectum (T3a-T3d)

**Fig. 1:**
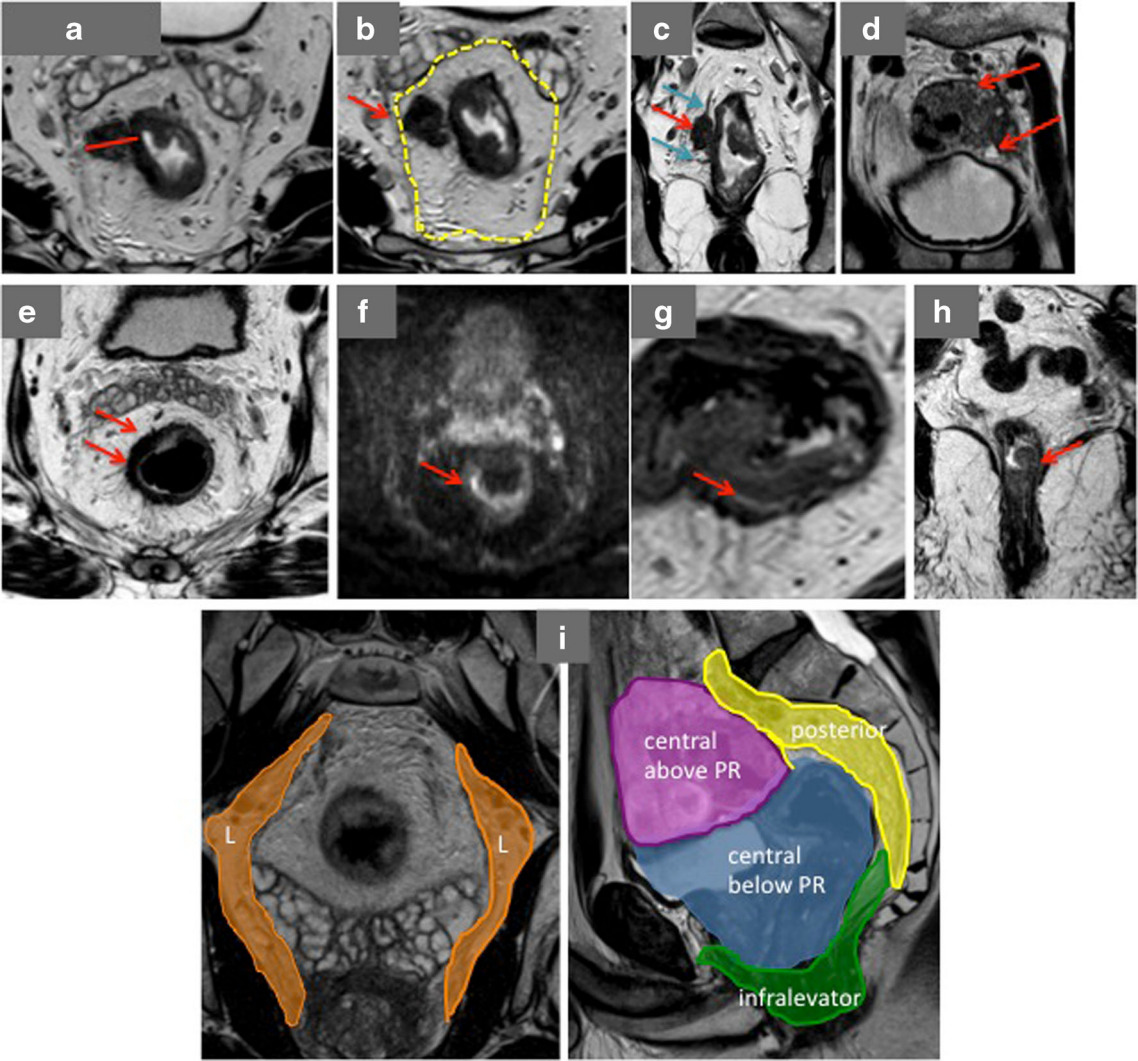
**a** An axial T2 image shows a semiannular tumour (before CRT) infiltrating rectal wall at 5–11 o’clock position. The extramural spread beyond the muscularis propria should be measured where it is the most advanced at 9–10 o’clock (*red line*). **b** A distance of less than 1 mm to the mesorectal fascia (*yellow line*) is considered as mrCRM + ve (*red arrow*). **c** A nodular deposit (*red arrow*) discontiguous with the rectal wall is located along the extramural veins (*blue arrow*). The extramural veins are also expanded and have intermediate signal within them—features of EMVI. **d** High signal intensity areas (*red arrows*) within the tumour are suggestive of mucinous content. **e** Low-density fibrosis is noted at the level of the treated tumour (*red arrows*) after CRT (the same patient as at Fig. 1a). **f** The same level of tumour as in Fig. 1e. High *b* value (1000) DW image shows evidence of restricted diffusion (*red arrow*); despite these findings, the patient has been enrolled in the deferral of surgery trial and is disease free for 3 years. **g** High-resolution axial MRI shows a sessile lesion infiltrating rectal wall at 4–6 o’clock position. A high signal intensity line (*red arrow*) is visible between the tumour and muscularis propria, which represent partially preserved submucosal layer. **h** A low rectal tumour confined to the part thickness of muscularis propria, indicating that the intersphincteric or mesorectal planes are safe. **i** Pelvic compartments are marked on these two high-resolution MR images (*L*—left compartment, central above the peritoneal reflection (*PR*), central below the *PR*, posterior, infralevator)

The depth of spread beyond the muscularis in millimetres has been proven to influence survival rates and is considered as an important prognostic factor that enables precise prognostic stratification within the T3 subcategory [13, 14]. Based on the Erlangen registry of 853 patients it has been shown to influence on a 5-year cancer specific survival [15, 16]. In 1993, Professor Hermanek of the Erlangen study group proposed dividing T3 stage into four sub-categories: T3a—spread beyond muscularis propria (MP) no greater than 1 mm; T3b—1–5 mm beyond the muscularis propria; T3c—5–15 mm beyond the muscularis propria; T3d—greater than 15 mm beyond the muscularis propria. All pathology studies to date that have assessed the depth of spread have shown this consistent relationship between extent of spread and prognosis that is independent of other pathological findings [15, 17, 18]. The MERCURY study group confirmed that high-resolution MRI measurement of depth of spread in millimetres showed very precise agreement with corresponding pathology measurements [19]. Furthermore the technique was able to differentiate low-risk patients for local recurrence when tumour spreads <5 mm into the mesorectum, negative mrCRM and mrEMVI against poorer prognostic group of patients with MRI-measured spread of tumour greater than 5 mm beyond the muscularis (Fig. 1a) [20, 21]. Thus recording depth of spread as T substage gives greater prognostic information than T and N stage when assessing rectal cancers using MRI.

### Extent of Colon Cancer Beyond the Muscularis Propria

Stratification of colon cancer according to the extramural spread beyond the muscularis propria has been proven to be useful method for identifying high risk patients who could benefit from neoadjuvant chemotherapy [22]. In a further study of 94 patients, Dighe et al. reported computed tomography (CT) to show 95 % sensitivity for differentiating T1/2 vs T3/T4 colon tumour [23]. Accuracy of radiological staging in 50 patients randomised within the pilot phase of the Foxtrot trial showed that 86 % (43 of 50) of tumours had adverse features (inoperable tumour, positive lymph nodes, EMVI or depth of invasion ≥5 mm) on pathological examination, predicting a greater than 50 % recurrence risk at 3 years. Preliminary results of the Foxtrot pilot has shown a likely benefit from the use of preoperative chemotherapy: a greater proportion of downstage tumours in pT0-T2 tumours (2 % in surgical group vs 9 % in the preoperative CT group of locally advanced colon cancers) and grade of tumour regression as moderate or more from 2 % vs 31 % (*p* < 0.0001) and a significantly lower rate of CRM involvement 4 % versus 20 % in favour of preoperative chemotherapy [24].

### Circumferential Margin

Whatever the depth of tumour infiltration at the level of infiltrating border assessment of the whole mesorectum is crucially important. Relationship of the tumour to the mesorectal fascia should always be reported. A distance on MRI of less than 1 mm to the mesorectal fascia was proven to predict pCRM status (Fig. 1b) [25]; moreover mrCRM on baseline scans was shown to be the most reliable prognostic factor for 5-year survival rates in MERCURY trial patients [26] and was more important than T and N stage. For low rectal tumours when almost no mesorectal fat serves as a boundary between tumour and external sphincter/levator relationship of the tumour to the intersphincteric plane is of the most importance [5••]. Even T2 tumours with full thickness invasion of the muscularis propria at the level of puborectalis sling should be considered as circumferential margin (CRM) plane threatened and for such patients a beyond TME plane extralevator abdominoperineal excision (APE) should be performed to reduce the risk of positive resection margin.

The mesorectum should also be assessed for presence of suspicious tumour deposits and extravascular tumour invasion; for tumours arising at a height of <6 cm from the anal verge, MRI detection of EMVI is independently associated with a risk of pathologic CRM involvement [5••].

### Lymph Nodes and Tumour Deposits

Published data suggest that morphological features such as heterogeneous signal intensity and lymph node irregular capsule border are accurate predictors of metastatic spread within the nodes [27, 28]. However, if good quality TME surgery is performed, nodal status seems to be of no prognostic importance for local recurrence [29, 30]. Shihab et al. reported that MR-identified lymph nodes involving mesorectal fascia are rarely a true cause of CRM infiltration on final pathology and that involvement of the CRM only by lymph nodes is uncommon [31].

Some nodular structures could represent vascular deposits, a phenomenon that is difficult to assess on final histology as absence of preserved nodular capsule preclude differentiating malignant lymph node with affected capsule from tumour deposit. However, the latter predicts poorer prognosis [32, 33]. High-resolution MRI allows differentiating EMVI and nodular deposits along the infiltrated veins (Fig. 1c), which are likely to represent venous deposits and could be classified according to TNM 7th edition as N1c (extranodal tumour deposits).

### MR EMVI

Both MRI and histopathologic EMVI are strong predictors of poor prognosis, particularly as a predictor of tumour metastasising to the liver [30]. In multivariate analysis, the presence of extramural vein invasion was significant for DFS [34, 35••]. Changes in the EMVI status before and after preoperative CRT from positive to negative improves the outcome (DFS): a 3-year DFS 87.8 % and 9 % recurrence against a 3-year DFS 45.8 % with 44 % recurrence (*p* < 0.0001) in those with no changes of EMVI status [36]. Survival outcomes of patients with stage II and III disease found that EMVI positive patients with stage II disease had similar outcomes to those patients with stage III disease. Histology seems to be less accurate in identifying EMVI especially after preoperative CRT [35••] and it has been stated by Royal College of Pathologists that EMVI is readily assessed by MRI (Fig. 1c) and this should be communicated to pathologists so that they can improve detection rates (especially following chemoradiotherapy) [37].

### Mucinous

Mucinous adenocarcinomas are associated with worse disease-free and overall survival outcomes compared to non-mucinous tumours and appear to be less sensitive to preoperative CRT when compared with non-mucinous tumours [6, 38]. Histopathology reports usually contain data about tumour differentiation; however, information about mucin presence within the tumour before patient undergoes surgery could be used in treatment decision-making. It has been shown that MRI enables visualisation of the mucinous component within the tumours, which appears as high signal intensity areas at the site of the tumour (Fig. 1d) and is of prognostic importance. Moreover, MRI pre-treatment diagnosis of mucinous tumour can be made more readily than preoperative histopathologic biopsy [39].

### Post CRT Tumour Response Assessment

#### Standard T2

##### MR RECIST and Volumetric Analysis

Different MRI techniques have been proposed as a tool for tumour response assessment; one of these is modified mr RECIST which is based on objective measurements of the change in craniocaudal length of measurable disease. According to new guidelines to evaluate the response to treatment in solid tumours, at least 30 % reduction rate should be considered as good or favourable response [40]. However, there is no established threshold for defining response in a luminal organ such as the rectum. MR volumetric analysis is another technique that was suggested to be a reliable tool for tumour response assessment [41, 42]. However there is again no published precise/accurate cutoff values of tumour volume reduction to predict favourable response or outcomes by comparing three dimensional tumour volumes of the tumour on pre- and post-CRT scans.

##### MR TRG

An MR regression-grading system based on the principles of the modified Mandard TRG system has been proposed for assessment of tumour response in rectal cancer classifying post-treatment changes. This divides response into five categories based on the proportion of intermediate tumour signal intensity versus low fibrosis signal intensity within the treated tumour (TRG1—total regression of the tumour which displays as liner low signal intensity scar on high resolution MRI scans; TRG2—low density fibrosis with no evident of macroscopic intermediate signal intensity within it (Fig. 1e); TRG3—MRI shows predominant fibrosis signal with some intermediate signal intensity areas; TRG4/5—predominantly intermediate signal intensity, minor regression, minimal fibrosis) [43]. In the retrospective CORE trial with 11 participant centres, different tumour regression parameters were assessed. Using binary logistic regression analysis it has been shown that only ymrT and MR TRG are significantly corresponds to final histology results compared to mrRECIST and mrVolume regression analysis [44]. Furthermore, MR TRG was shown to be an independent prognostic factor of disease-free survival [12].

#### Diffusion-Weighted Imaging

Published data suggests that an objectively useful role of diffusion imaging for primary rectal cancer assessment and restaging after chemoradiotherapy has not yet been found. Residual disease is expected when hyperintense signal is visualised on high *b* value diffusion-weighted images at the site of treated tumour (Fig. 1f) [45, 46]. However, there is no evidence showing that this technique was validated against other existing parameters and patient outcomes. It has been proposed that combined DWI and T2 WI could increase MRI accuracy for identifying complete responders; however, the results only significant for those radiologists with not much of experience in reading rectal MRIs [45]. Whereas inter-observer agreement for MR TRG was shown to be 0.8 regardless of experience suggesting this method to be reproducible [47]. Furthermore incorporating MR TRG into assessment of surgical plane safety in the low rectal cancer after chemoradiotherapy enabled good prediction of likely clear margins [5••].

## Defining Surgical Planes

### Early Rectal Cancer

Staging of early rectal tumours could be a challenge, mistakes in image interpretation lead to over or undertreatment of patients, who should on the contrary benefit from being diagnosed on the early stage of tumour invasion. For tumours less than T1 sm3 without any poor prognostic features (such as lymph nodes or extramural venous invasion) local excision could be considered as the main treatment option.

Since the 1990s, endorectal ultrasound (ERUS) has been considered as the best diagnostic modality for early rectal cancer, and accuracy rates were reported to be as high as 90 % [48–50]. However, more resent data suggests the contrary; Garcia-Aguilar et al. reported ERUS to be an inaccurate (unreliable) tool for T1 and T2 early rectal cancer differentiation (59 %), showing that recurrence and survival rates were not influenced by the fact patients having or not having preoperative ERUS [51]. Sailer M. et al. proposed that ERUS is of no help in the assessment of T2 carcinomas [52]. Accuracy of ERUS in detection lymph node metastases ranges from 60 to 80 %, so that preoperative ERUS staging cannot exclude node positive status [53, 54].

In our study where treatment decision was made by clinical not mr-assessment [55], MRI accuracy of identifying patients eligible for organ-preserving surgery (tumours less than T1sm3) was 73 %. If the decision had been made based on MRI, T stage TME surgery vs local excision could have been offered in significantly fewer patients—14 % (6/43) (*p* < 0.01). The presence of at least 1 mm of submucosal layer identified as high signal intensity line between the tumour-advancing edge and muscularis suggest that LE is safe and feasible to perform (Fig. 1g).

### Low Rectal Tumours

Distal tumour margin lower or at the level of 6 cm from the anal verge indicates a low-lying tumour in the portion of the mesorectum that tapers and is thus at higher risk of margin involvement during TME plane surgery. Recurrence free survival improves with an increased likelihood of receiving the appropriate treatment when low rectal tumours are preoperatively staged by MRI [56, 57]. High-resolution MRI enables staging and assessment of the surgical planes in patients with low rectal cancer when the relationship to the intersphincteric plane, external sphincter and levators are of the main concerns.

In low rectal tumours confined to the submucosal layer/part thickness of muscularis propria, the intersphincteric or mesorectal planes are safe and intersphincteric APE or ultra low TME is possible (Fig. 1h).

When tumour extends through the full thickness of the muscularis propria, into the intersphincteric plane or into the external sphincter, full clearance is unlikely, and therefore an extralevator APE is indicated for radial clearance.

### Beyond TME

In patients with CRM involvement, R0 resection can only be achieved if surgical planes are extended beyond the TME plane, and the radiologist plays a fundamental role in both identifying these cases and correctly assessing the surgical planes required for histological clearance. High-resolution T2-WI MR allows identifying surgical planes and indicates when extralevator abdominoperineal resections (ELAPE) and exenterative surgery could potentially be performed [58]. On multivariate analysis, only a positive resection margin was a significant predictor of reduced local recurrence free survival (hazard ratio, 5.48; *p* = 0.002) [59].

A useful system is to divide the pelvis into six surgical compartments (central above peritoneal reflection: any structure above the peritoneal reflection/uterus; anteriorly below peritoneal reflection: bladder/upper vagina/ovaries/prostate/seminal vesicles/urethra; posteriorly: bony cortex/periosteum S1-5, coccyx/presacral fascia (S1-5)/sciatic nerve/sacral nerve branches (S1/2); laterally: pelvic fascia/pelvic sidewall/internal/external iliac vessels/sacrotuberous/sacrospinous ligaments/piriformis/obturator internus muscles; infralevator: Levator muscles/sphincter complex; anterior urogenital triangle/perineum: vaginal introitus/urethra/retropubic space) [58] (Fig. 1i).

## Conclusion

Treatment decisions are no longer made by a single specialist but a colorectal unit team, when pre-treatment tumour spread assessment drives the type of treatment to be chosen. Imaging plays a pivotal role in guiding the treatment management. Surgeons push forward radiologists to improve staging accuracy of most accepted tumour spread criteria such as T and N; however, the paradigm of tumour spread prognostic factors has shifted and new imaging biomarkers are emerging into the routine radiological practise.

Such tumour spread markers as EMVI, relationship to the mesorectal fascia and intersphincteric plane (CRM status), presence of mucin component within the tumour have a greater influence on patient outcomes than T and stage and therefore should now be factored into treatment decisions.

Imaging enables better stratification of patients in risk groups for local and distal failure, helps to clarify safe surgical planes, identifies those patients who could potentially benefit from preoperative treatment and ultimately could identify those patients for whom surgery may be safely omitted of deferred.
